# The Effect of Atrial Fibrillation on Inpatient Outcomes of Patients with Acute Pancreatitis: A Two-year National Inpatient Sample Database Study

**DOI:** 10.19102/icrm.2020.111205

**Published:** 2020-12-15

**Authors:** Shakeel Jamal, Muhammad Zatmar Khan, Asim Kichloo, Ehizogie Edigin, Beth Bailey, Michael Aljadah, Ishtiaq Hussaian, Asad Ur Rahman, Muhammad Ahmad, Khalil Kanjwal

**Affiliations:** ^1^Department of Internal Medicine, St. Mary’s of Michigan, Saginaw, MI, USA; ^2^Central Michigan University College of Medicine, Saginaw, MI, USA; ^3^John H. Stroger Hospital of Cook County, Chicago, IL, USA; ^4^Medical College of Wisconsin Affiliated Hospitals, Milwaukee, WI, USA; ^5^Department of Gastroenterology, Cleveland Clinic Florida, Weston, FL, USA; ^6^McLaren Greater Lansing Hospital, Lansing, MI, USA

**Keywords:** Atrial fibrillation, National Inpatient Sample, pancreatitis, outcomes

## Abstract

Limited published data exist regarding the association of atrial fibrillation (AF) and acute pancreatitis. To test our hypothesis that AF increases mortality and clinical outcomes in patients with acute pancreatitis, we conducted a cross-sectional data review of the National Inpatient Sample (NIS) database. The NIS database was reviewed for the collection of data on patient hospitalizations in 2016 and 2017. Patients diagnosed with acute pancreatitis with and without concomitant AF were included in the analysis. The International Classification of Diseases, 10th revision coding system was used for the variables of interest. The Stata software program (StataCorp LLC, College Station, TX, USA) was used to perform statistical analyses. The chi-squared test or analysis of variance was used to identify differences in demographic characteristics between the groups. The study population included two groups of patients: those with acute pancreatitis only (n = 542,440) and those with both acute pancreatitis and AF (n = 32,790). The group with acute pancreatitis and AF had a two- to threefold higher rate of mortality [adjusted odds ratio (OR): 2.59; 95% confidence interval (CI): 2.04–3.28] and increased length of stay (adjusted OR: 1.28; 95% CI: 1.08–1.48). Also, significantly higher odds of sepsis (adjusted OR: 2.49; 95% CI: 2.06–3.01), congestive heart failure (adjusted OR: 3.16; 95% CI: 2.87–3.49), acute coronary syndrome (adjusted OR: 1.61; 95% CI: 1.17–2.21), stroke (adjusted OR: 3.94; 95% CI: 1.42–10.93), and acute kidney injury (adjusted OR: 1.42; 95% CI: 1.30–1.55) were observed in patients with acute pancreatitis and AF relative to in patients with acute pancreatitis only. Our results suggest AF increases mortality in patients with acute pancreatitis and that patients with acute pancreatitis and AF are at greater risk of worse clinical outcomes.

## Introduction

Acute pancreatitis is an inflammatory condition involving the pancreatic and/or peripancreatic tissues. It can lead to systemic inflammatory response syndrome (SIRS) and, if severe, can result in multiorgan failure.^1–6^ Mortality in acute pancreatitis can occur in two waves: an early wave due to underlying SIRS and multiorgan dysfunction syndrome (MODS) and a late wave attributed to MODS combined with sepsis caused by pancreatic tissue necrosis due to infection.^7^ Survival is determined by the early development of SIRS and the persistence of MODS.^8^

When MODS occurs, the cardiovascular system may be affected, which results in cardiac rhythm and contractility disturbances as well as peripheral vasomotor dysfunction. Changes in cardiac physiology have been reported at the histological level, with myofiber edema and disruption of the intercellular junctions.^4,5^ These changes at the cellular level can result in hemodynamic, cardiac conduction, and pericardial abnormalities.

Acute pancreatitis has an incidence ranging from five to 80 per 100,000 people.^6^ Acute pancreatitis is the most common cause of gastrointestinal-related hospital admission.^8^ The mortality of hospitalized patients with acute pancreatitis is 5% to 10% and up to 30% in severe cases.^9,10^ Several different scoring systems have been developed to predict in-hospital mortality in acute pancreatitis based on several clinical variables. Studies have reported an association of acute pancreatitis with atrial fibrillation (AF) and flutter that resolves after treatment of acute pancreatitis.^11^ We hypothesized that AF may worsen the clinical outcomes in patients with acute pancreatitis and that good control of AF may improve patient outcomes. To further evaluate our hypothesis, we conducted a cross-sectional analysis.

## Methods

### Data source

The NIS has been elaborated on in detail in a prior study.^12^ Briefly, it is the largest publicly available database in the United States that falls under the purview of the Healthcare Cost and Utilization project and is maintained by the Agency for Healthcare Research and Quality. It is one of the most useful databases used to assess outcomes and trends of various procedures and diseases and includes de-identified data collected from 20% of community hospitals in 46 states. Each hospitalization is representative of one primary diagnosis, up to 29 secondary diagnoses, and 15 procedures using International Classification of Diseases (ICD), ninth revision, clinical modification (CM) or ICD, 10^th^ revision (ICD-10)-CM codes. The data include admission status, demographics, admitting diagnosis, comorbidities, location of health-care facility (rural or urban), discharge diagnosis, outcomes, length of stay, and cost during hospitalization. We examined all adult patients who were hospitalized in the United States in 2016 and 2017 with the diagnosis of acute pancreatitis and comorbid AF using the NIS. Patients were filtered using ICD-10-CM codes.

We identified all adult patients older than 18 years who were admitted with acute pancreatitis with and without concomitant primary or secondary AF in 2016 and 2017. Our institution does not require the attainment of ethical approval for NIS database studies. We excluded any patients who were hospitalized with missing demographics (eg, age, sex, admission or discharge diagnosis, and mortality data). We used NIS variables to identify patients’ age, sex, race, county location, county income, number of hospital beds, and alcohol abuse. Race was divided into two categories (white and nonwhite).

### Outcomes

Our objectives were to assess the disease severity and inpatient outcomes in patients admitted with a principal diagnosis of acute pancreatitis with and without concurrent AF. The primary outcomes analyzed were mortality of all patients admitted with a principal diagnosis of acute pancreatitis with and without concurrent AF. Secondary outcomes associated with acute pancreatitis and AF were heart block, cardiogenic shock, cardiac arrest, sepsis, hemorrhage, stroke, heart failure, acute coronary syndrome, pericardial complications, cardiac electronic implantable devices, valvular complications, deep venous thrombosis and pulmonary embolism, acute kidney injury, acute kidney injury requiring hemodialysis, and length and cost of hospital stay.

### Statistical analysis

We used survey analyses for stratifying and clustering encounters for all continuous and categorical variables. The Stata software program (StataCorp LLC, College Station, TX, USA) was used to perform statistical analyses. We used the chi-squared test or analysis of variance to identify differences in categorical variables and the two-sample t-test for the analysis of continuous variables. A logistic regression model was used to calculate the odds ratio (OR) for outcomes between the two study groups. This was followed by the conduct of multivariate analyses to account for any confounders in the form of comorbidities between the two groups. A p-value of less than 0.05 was considered to be statistically significant. We audited the analyses using the checklist provided by the NIS to assess and ensure that the data analyses were compliant with the rules recommended by the NIS.^13^ Multivariate analysis was performed and adjusted for statistically significant baseline characteristics (eg, age, sex, race, Charlson Comorbidity Index, hospital bed size, primary payer, median household income, hospital region, dyslipidemia, old myocardial infarction, old percutaneous intervention, old coronary artery bypass grafting, history of pacemaker implantation, chronic obstructive pulmonary disease, carotid artery disease, history of stroke, hypertension, peripheral arterial disease, diabetes mellitus types 1 and 2, obesity, chronic kidney disease, liver disease, and history of smoking).

## Results

We identified a total of 71 million hospitalizations in 2016 and 2017. Of these, we further identified 575,230 patients with a principal diagnosis of acute pancreatitis based on the ICD-10 codes mentioned in **Table 1**. Of these, 542,440 patients did not have concurrent AF and 32,790 patients had concurrent AF. Thus, our final sample included two study groups: patients with acute pancreatitis only and patients with acute pancreatitis and AF. **Table 2** shows the background characteristics by study group; patients with acute pancreatitis and AF were significantly older (70.47 versus 50.78 years; p < 0.0001), more often were male (42% versus 47%; p < 0.001), more frequently were white (79% versus 63%; p < 0.0001), and had Charlson Comorbidity Index scores of two or three or more points (p < 0.0001) than those participants in the group with acute pancreatitis only. Patients with acute pancreatitis and AF had higher baseline comorbidity burdens such as dyslipidemia, old myocardial infarction, percutaneous coronary intervention and coronary artery bypass graft, coronary artery disease, chronic obstructive pulmonary disease, peripheral arterial disease, hypertension, diabetes mellitus types 1 and 2, obesity, chronic kidney disease, liver disease, maintenance hemodialysis, oxygen dependence, and anemia.

**Table 3** summarizes the results of the logistic regression analyses of the adjusted ORs used to control for the variables in **Table 2**. Patients with acute pancreatitis and AF demonstrated twofold to threefold greater mortality [adjusted OR: 2.59; 95% confidence interval (CI): 2.04–3.28]. Patients with acute pancreatitis and AF also had higher odds of heart block (adjusted OR: 1.86; 95% CI: 1.55–2.24), cardiac arrest (adjusted OR: 2.68; 95% CI: 1.76–4.09), cardiogenic shock (adjusted OR: 3.14; 95% CI: 1.39–7.09), pericardial complications (adjusted OR: 3.38; 95% CI: 2.21–5.17), valvular complications (adjusted OR: 1.76; 95% CI: 1.49–2.08), acute coronary syndrome (adjusted OR: 1.61; 95% CI: 1.17–2.21), cardiac implantable electronic devices (adjusted OR: 9.52; 95% CI: 3.79–23.94), stroke (adjusted OR: 3.94; 95% CI: 1.42–10.93), congestive heart failure (adjusted OR: 3.16; 95% CI: 2.87–3.49), acute kidney injury (adjusted OR: 1.42; 95% CI: 1.30–1.55), acute kidney injury requiring hemodialysis (adjusted OR: 2.55; 95% CI: 1.17–5.60), sepsis (adjusted OR: 2.49; 95% CI: 2.06–3.01), increased length of hospital stay (adjusted OR: 1.28; 95% CI: 1.08–1.48), and greater cost of hospital stay (adjusted OR: 17,489; 95% CI: 14,349–20,628).

## Discussion

The principal findings of this study were the following: there was a significantly increased mortality rate among patients with acute pancreatitis and concurrent AF, the cardiovascular morbidity burden was significantly higher in acute pancreatitis and AF, patients with acute pancreatitis and AF were at significantly increased risk of stroke, concurrent AF increases the risk of renal failure in patients, and age and male sex are important and significant predictors of outcomes in patients with acute pancreatitis and AF.

Mortality in acute pancreatitis depends on the disease severity and varies from 2% in mild cases to 46% in the most severe context.^14^ This is thought to result from MODS.^15^ Similarly, AF is known to cause a fourfold increased mortality risk relative to that in the general population.^16^ AF is an independent risk factor for mortality after adjusting for cardiovascular comorbidities.^17^ We report a statistically significant twofold to threefold increase in mortality with an adjusted OR of 2.59 in patients with acute pancreatitis and concurrent AF. This trend is thought to be compounded by an increase in other adverse outcomes such as acute coronary syndrome, congestive heart failure, cardiac arrest, heart blocks, cardiogenic shock, stroke, and sepsis. Persistent organ dysfunction can result in a mortality rate as high as 30%.^18^

The outcomes of acute pancreatitis depend on its severity, ranging from self-limiting episodes to multiorgan failure leading to mortality.^19^ This is related to the degree of insult to the pancreas, which can be mild, in the form of interstitial edematous pancreatitis, or more severe, such as necrotizing pancreatitis, which warrants surgical intervention.^20,21^ In one study, alcohol was considered as a major triggering factor, followed by gallstones, iatrogenic factors, and hyperlipidemia. In the same study, pulmonary, cardiovascular, renal, hematologic (disseminated intravascular coagulation), and hepatic complications were primarily reported as elements of MODS, whereas cardiac failure was the major cause of mortality in both the in-patient setting and during follow-up.^22^ In another retrospective study, the prevalence rates of cardiovascular comorbidities, including heart failure, coronary artery disease, and AF, were higher among patients with chronic pancreatitis.^23^

Acute pancreatitis can manifest as SIRS, which is considered to be an important prognostic factor in determining the morbidity and mortality rates.^24,25^ SIRS triggers the release of inflammatory cytokines (tumor necrosis factor–α, interleukin-6, and C-reactive protein), leading to multiorgan failure, which, in turn, places increased stress on the heart and triggers malignant arrhythmias.^26^ AF is considered one of the most commonly observed arrhythmias in critically ill and hospitalized patients.^27^ The possible mechanisms may include superimposed effects of fluid resuscitation, which is the mainstay of management to control inflammation, and hemodynamic support, leading to volume overload in patients with cardiovascular-compromised states (eg, heart failure, ischemic heart disease), resulting in atrial myocardial stretch and triggering AF. Another possible mechanism could be an imbalance between the sympathetic and parasympathetic nervous systems, with changes in hemodynamics triggering alterations in heart rate variability, resulting in atrial and ventricular arrhythmias.^27–30^

AF is considered an important prognostic factor in determining morbidity and mortality in both critical and noncritical patients due to the risk of thromboembolism and stroke, impairment in the quality of life, congestive heart failure, pulmonary/renal complications, and increased health-care costs.^31–35^ Severe forms of acute pancreatitis can result in sepsis and septic shock, which, if they cause new-onset AF, are known to culminate in adverse outcomes such as a prolonged and increased need for mechanical ventilation, longer hospital/intensive care unit length of stay, and increased mortality (69% versus 40%) as compared with among patients without AF.^33^ The prevalence of sepsis in the group with acute pancreatitis and AF was 4.06% as compared with 1.19% in the group with acute pancreatitis only, which could very well explain the possibility of new-onset AF.

The purpose of this study was to show and quantify how concurrent AF worsens the disease burden and mortality in patients admitted in an inpatient setting for acute pancreatitis. We have observed an increased risk of cardiovascular, neurologic, and renal complications in patients with acute pancreatitis and concurrent AF as compared with among patients with acute pancreatitis only. These complications lead to additional interventions that contribute to the increased length of stay. The mean difference in the length of stay between the two groups in our study was approximately two days.

## Limitations

The inherent nature of a cross-sectional study did not allow us to calculate the incidence and rate ratios. The use of the Healthcare Cost and Utilization project database also has limitations of its own—for example, with respect to the selected group of patients included in the database. We could not stratify acute pancreatitis according to severity based on the gathered data, which can have prognostic implications. Similarly, it could not be determined whether the patients had paroxysmal or persistent AF. Meanwhile, the NIS database is primarily compiled based on ICD codes, which represent “claims data” for reimbursement purposes and for summarizing clinical presentations in retrospect; it does not give us information regarding the clinical presentation (ie, “clinical data”). Further studies are required to incorporate both claims and clinical data to ensure a deeper understanding of outcomes in patients admitted for acute pancreatitis and to assess its outcomes while influenced by the presence of concurrent AF. The other limitation of multivariate regression analysis is that it cannot determine causation; only the association between predictors and outcomes can be revealed. However, similar to propensity matching, multivariate regression analysis can also be used to adjust for confounders and differences in baseline characteristics between two cohorts.

Separately, this study has some strengths if carefully analyzed. The large sample size, which was made possible with the use of the NIS database, increases the precision of the study results and also allowed us to gather information about other variables and comorbidities, which have provided meaningful results. The increased mortality rate and significant trend toward other outcomes in the group of patients with pancreatitis and AF can serve as a platform for further research. Given the increased prevalence of these two comorbidities worldwide, new ideas and management strategies should be formulated, which can alter the future of clinical practice for good.

## Conclusion

The presence of concurrent AF in patients with acute pancreatitis predicts higher mortality rates and adverse clinical outcomes. Further studies in the future can help us to understand the underlying pathogenesis of these outcomes and also help with devising strategies to improve outcomes in such a cohort of patients.

## Figures and Tables

**Table 1:** ICD-10-CM and Procedure Coding System

Variable	ICD 10 Codes
Acute pancreatitis	K85.0, K85.00, K85.01, K85.02, K85.1, K85.10, K85.11, K85.12, K85.2, K85.20, K85.21, K85.22, K85.3, K85.30, K85.31, K85.32, K85.8, K85.80, K85.81, K85.82, K85.9, K85.90, K85.91, K85.92
Atrial fibrillation/flutter	I48, I48.0, I48.1, I48.11, I48.19, I48.2, I48.20, I48.21, I48.3, I48.4, I48.9, I48.91, I48.92
Bleeding complications	K29.01, K62.5, K31.811, K57, K29, K25, K26, K27, K28, I85.01, N93
Heart failure	I501, I5020, I5021, I5022, I5023, I5030, I5031, I5032, I5033, I5040, I5041, I5042, I5043, I50810, I50811, I50812, I50813, I50814, I5082, I5083, I5084, I5089, I509
Heart block	I445, I452, I4430, I441, I447, I455, I444, I442, I440, I4439, I4460, I4469, I450, I4510, I4519, I453, I454
Valvular heart diseases	I340, I341, I342, I348, I349, I350, I351, I352, I358, I359, I360, I361, I362, I368, I369, I370, I371, I372, I378, I379
Cardiogenic shock	R57.0
Stroke	I69.30, I69.31, I69.320-I69.323, I69.328, I69.331-I69.334, I69.339, I69.341-I69.344, I69.349, I69.351-I69.354, I69.359, I69.361-I69.364, I69.369, I69.390-I69.393, I69.398, I63, I69, I6010, I6011, I6012, I602, I6030, I6031, I6032, I604, I6050, I6051, I6052, I606, I607, I608, I609, I610, I611, I612 I613, I614, I615, I616, I618, I619, I6200, I6201, I6202, I6203, I621, I629, I60
In-hospital cardiac implantable electronic device placement	0JH604Z, 0JH634Z, 0JH804Z, 0JH834Z, 0JH605Z, 0JH635Z, 0JH805Z, 0JH835Z 0JH606Z, 0JH636Z, 0JH806Z, 0JH836Z 0JH60PZ, 0JH63PZ, 0JH80PZ, 0JH83PZ 0JH608Z, 0JH638Z, 0JH808Z, 0JH838Z
Cardiac arrest	I46, I468, I469, I462
In-hospital resuscitation	5A2204Z
Acute coronary syndrome	I2101, I2102, I2109, I2111, I2119, I2121, I2129, I213, I214, I219, I21A9, I220, I221, I222, I228, I229
Acute kidney injury	N170, N171, N172, N178, N179
Hemodialysis	5A1D70Z, 5A1D80Z, 5A1D90Z
Postprocedural pneumothorax	J95.811
Postoperative respiratory failure	J95.821
Other iatrogenic respiratory complications	Ventilator-associated pneumonia (J95.851) Postprocedural aspiration pneumonia (J95.89) Other respiratory complications (J95.859, J95.88, J95.89)
Sepsis	A40.0, A40.1, A40.8, A40.9, A40.3, A41.89, A41.4, A41.59, A41.81, A41.1, A41.3, A41.9, A41.01, R65.21, R65.20, R65.10, R65.11

**Table 2:** Baseline Characteristics of Acute Pancreatitis Hospitalizations with and without AF (n = 575,230)

	Without AF (n = 542,440)	With AF (n = 32,790)	p-value
Mean age, years	50.78	70.47	< 0.0001
Female sex	47.18%	42.05%	< 0.0001
Race			< 0.0001
White	63.49%	79.78%	< 0.0001
Black	17.53%	9.76%	< 0.0001
Hispanic	13.12%	6.15%	< 0.0001
Asians	2.05%	1.94%	0.003
Native Americans	0.89%	0.6%	< 0.0001
Others	2.92%	1.77%	< 0.0001
Charlson Comorbidity Index			< 0.0001
0	42.15%	19.17%	
1	30.22%	23.35%	
2	13.45%	19.64%	
≥3	14.18%	37.85%	
Hospital bed size			< 0.0001
Small	23.43%	21.53%	
Medium	30.83%	29.46%	
Large	45.74%	49.01%	
Hospital teaching status			0.1066
Nonteaching	40.10%	39.02%	
Teaching	59.90%	60.98%	
Hospital location			0.5569
Rural	12.18%	11.92%	
Urban	87.82%	88.08%	
Expected primary payer			< 0.0001
Medicare	29.63%	72.10%	
Medicaid	26.28%	9.0%	
Private	34.36%	16.53%	
Self-pay	9.73%	2.36%	
Median household income (quartile)			< 0.0001
1^st^ (0–25^th^)	33.26%	29.16%	
2^nd^ (26th-50^th^)	26.67%	27.29%	
3^rd^ (51st-75^th^)	23.12%	24%	
4^th^ (76th-100^th^)	16.95%	19.56%	
Hospital region			0.0061
Northeast	16.73%	18.18%	
Midwest	22.55%	23.38%	
South	41.03%	39.45%	
West	19.69%	19.00%	
Dyslipidemia	31.49%	49.85%	< 0.0001
Old MI	3.26%	9.94%	< 0.0001
Old PCI	0.33%	1.14%	< 0.0001
Old CABG	2.08%	9.18%	< 0.0001
Old pacemaker	0.71%	8.75%	< 0.0001
COPD	8.90%	20.62%	< 0.0001
Carotid artery disease	0.17%	0.91%	< 0.0001
HTN	46.56%	51.45%	< 0.0001
Peripheral vessel disease	1.31%	4.82%	< 0.0001
Hypothyroidism	8.32%	16.74%	< 0.0001
DM types 1 and 2	25.93%	34.35%	< 0.0001
Obesity	15.85%	18.15%	< 0.0001
CKD	8.03%	24.53%	< 0.0001
Liver disease	19.10%	14.62%	< 0.0001
Electrolyte imbalance	31.92%	34.55%	< 0.0001
Maintenance hemodialysis	1.57%	3.05%	< 0.0001
O_2_ dependence	0.79%	3.03%	< 0.0001
Smoking	19.14%	27.08%	< 0.0001
Anemia	16.90%	24.87%	< 0.0001
Taking anticoagulants	2.07%	33.36%	< 0.0001
CAD	8.91%	32.02%	< 0.0001

AF: atrial fibrillation/flutter; CABG: coronary artery bypass graft; CAD: coronary artery disease; CKD: chronic kidney disease; COPD: chronic obstructive pulmonary disease; DM: diabetes mellitus; HTN: hypertension; median household income: median household income for patient’s zip code; MI: myocardial infarction; O_2_: oxygen; PCI: percutaneous coronary intervention.

**Table 3:** Clinical Outcomes of Acute Pancreatitis with and without AF

	Acute Pancreatitis Without AF (n = 32,790), %	Acute Pancreatitis and AF (n = 542,440), %	Adjusted OR (95% CI)	p-value
Primary outcome				
In-hospital mortality	0.46	2.76	2.59 (2.04–3.28)	< 0.0001*
Secondary outcomes				
Heart block	0.81	4.09	1.86 (1.55–2.24)	< 0.0001*
Cardiogenic shock	0.04	0.32	3.14 (1.39–7.09)	0.006*
Cardiac arrest	0.19	0.81	2.68 (1.76–4.09)	< 0.0001*
Pericardial complications	0.15	0.76	3.38 (2.21–5.17)	< 0.0001*
Valvular complications	0.87	5.31	1.76 (1.49–2.08)	< 0.0001*
Bleeding complications	11.51	13.40	0.92 (0.84–1.01)	0.073
ACS	0.31	1.52	1.61 (1.17–2.21)	0.003*
Stroke	0.02	0.15	3.94 (1.42–10.93)	0.009*
CHF	4.17	28.77	3.16 (2.87–3.49)	< 0.0001
CIED	0.01	0.20	9.52 (3.79–23.94)	< 0.0001*
Sepsis	1.19	4.06	2.49 (2.06–3.01)	< 0.0001*
AKI	10.1	23.83	1.42 (1.30–1.55)	< 0.0001
AKI requiring HD	0.04	0.21	2.55 (1.17–5.60)	0.019*
DVT/PE	1.14	2.27	0.88 (0.66–1.16)	0.350
Adjusted mean difference				
Mean LOS, days	4.22	6.13	1.28 (1.08–1.48)	< 0.0001*
Mean total charge, USD	36,980	59,631	17,489 (14,349–20,628)	< 0.0001*

ACS: acute coronary syndrome; AF: atrial fibrillation/flutter; AKI: acute kidney injury; CHF: congestive heart failure; CI: confidence interval; CIED: cardiovascular implantable electronic device; DVT: deep venous thrombus; HD: hemodialysis; LOS: length of hospital stay; OD: odds ratio; PE: pulmonary embolus; USD: United States dollars.

*Statistically significant after adjusting for the variables in **Table 2**.
